# The Impact of MEX 3D Printing Key Control Settings on the Rheology and DMA Response of Bacteria-Derived PHA

**DOI:** 10.1021/acsomega.5c04386

**Published:** 2025-06-23

**Authors:** Markos Petousis, Nikolaos Michailidis, Nikolaos Mountakis, Apostolos Argyros, Maria Spyridaki, Emmanuel Maravelakis, Nektarios Nasikas, Nectarios Vidakis

**Affiliations:** † Department of Mechanical Engineering, 112178 Hellenic Mediterranean University, Heraklion 71410, Greece; ‡ Physical Metallurgy Laboratory, Mechanical Engineering Department, School of Engineering, 37782 Aristotle University of Thessaloniki, 54124 Thessaloniki, Greece; § Centre for Research & Development of Advanced Materials (CERDAM), Center for Interdisciplinary Research and Innovation, Balkan Centre, Building B’, 10th km Thessaloniki-Thermi road, 57001 Thessaloniki, Greece; ∥ Department of Electronic Engineering, Hellenic Mediterranean University, Chania 73133, Greece; ⊥ Division of Mathematics and Engineering Sciences, Department of Military Sciences, 69139 Hellenic Army Academy, 16673 Vari, Attica, Greece

## Abstract

The wide range of materials featuring unique properties has contributed to the constant growth of 3D-printed items nowadays. Polyhydroxyalkanoate (PHA) is a biosourced material that is gradually growing in additive manufacturing. 3D printed PHA was examined herein under dynamic mechanical analysis. The aim was to reveal the critical 3D printing settings affecting the response of this eco-friendly polymer on its rheology and under combined thermal and force loadings, producing valuable information for the enrichment of the available experimental data. Optimization was attempted with Taguchi L9 experimental design, with four control parameters: deposition speed, layer height, extrusion temperature, and extrusion width. The response metrics were the Flexural Storage Modulus, Dynamic Glass Transition Temperature, and Damping Factor at Dynamic Glass Transition Temperature. Two regression models were applied and compared to form reliable prediction equations, and a confirmation run verified the outcome. Optical microscopy evaluated the samples’ microstructure and quality. Two controls were distinguished for their remarkable impact, namely, deposition speed and layer height. Flexural Storage Modulus increased ∼15% with optimized settings selection. The optimization significance is unequivocal, promoting the utilization of PHA in Additive Manufacturing, with the valuable information provided on the mechanical response of this nature-sourced polymer.

## Graphical abstract

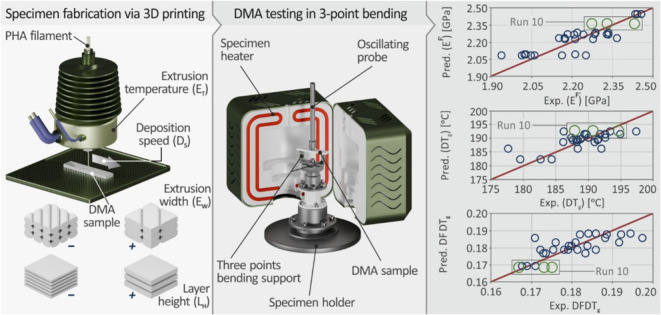

## 1 Introduction

Additive Manufacturing (AM) is a processing procedure applicable to a variety of materials such as polymers,^1^ metals,^2^ and ceramics.^3,4^ AM can provide customization, shape complexity, short time, and reduced cost.^5,6^ Conventional polymers are an attractive option in 3D printing AM. Still, there is also a tendency to introduce biobased or biodegradable polymers to research and industrial fields to promote eco-friendliness. This requires dealing with their processing challenges.^7^ Their contribution to sustainable development^8,9^ makes them desirable and attractive choices that could potentially replace other less sustainability-supportive materials.

Such materials are typically poly­(lactic acid) (PLA)^10^ or polyhydroxyalkanoates (PHAs).^11^ In AM, PLA is among the most popular thermoplastics. It is thoroughly investigated for its mechanical properties,^12,13^ its sustainability,^14^ and its performance as a matrix in composites with various types of additives.^15–17^ Research aims among others, to improve its eco-friendliness. On the other hand, PHA research in AM is still limited, with its nature-sourced characteristics concealing additional challenges. Thermoplastic PHAs are polyesters of hydroxyalkanoic acids that can be biotechnologically created. They are based on bacteria,^4^ as well as extremophilic archaea fermentation,^18,19^ formed in an aqueous environment.^20,21^ They are characterized as biobased, biodegradable, nontoxic, and available from renewable resources.^22–24^ They can biodegrade in many environments and be an alternative to single-use plastics and those that cannot be repurposed in the manufacturing sector because of their poor quality when recycled.^25^ Some microorganisms that can contribute to PHAs production include *Escherichia coli*,^26,27^ Pseudomonas,^28^ Aeromonas,^29^ Azotobacter,^30^ Cupriavidus,^31^ Clostridium,^32^ Methylobacterium,^33^ Ralstonia,^34^ and Syntrophomonas.^35^

PHAs have a wide range of applications, including biomedicals,^36–38^ agriculture,^39,40^ electronics,^41^ sensors,^42,43^ and packaging.^44–51^ They can be employed in cases where 3D-printing is utilized; however,^52–54^ they have not been expanded significantly yet. PHA has mostly been found to be combined with PLA in various studies.^55–57^ PLA/PHA biodegradable polymer blends have previously been investigated for process parameters’ optimization in AM 3D printing.^58^ The properties of the manufactured coupons were investigated to determine the most effective set of printing parameters that would result in the best performance under tensile and compression loadings in ambient room conditions. In an additional investigation, PHA was again used as a reinforcement for PLA to improve the impact strength of 3D printed samples.^59^

There are challenges that accompany the 3D printing procedure of various polymeric materials, especially with respect to the complexity of discovering the most beneficial set of printing parameters that are suitable for each material. This issue can be confronted by employing optimization techniques and an appropriate design of the experiment.^60–63^ Consequently, the experimental procedure is more stable and focuses on adjusting and examining the most important points of the investigation, resulting in production efficiency.^64^ Some of the most well-known designs of experiments are the Taguchi design,^65^ Box-Behnken,^66–69^ design, and full factorial design.^70–73^ PHA has not been utilized in combination with the aforementioned methods, except in some cases where it is combined with other materials.^58,74^ However, there are plenty of other materials, such as PLA,^75–78^ ABS,^79^ PC,^80^ PA6,^68,81^ PEEK,^82,83^ PEI,^84^ PCL,^85^ PMMA,^86,87^ and ASA,^88^ which can be found in the literature, that are analyzed and optimized with various experimental design methods.

Despite the limited growth of PHA for the time being, its market size seems to have the potential for great increase, judging by the various available reports estimating the PHA market value.^89–93^ In particular, according to a report provided by Grand View Research,^94^ it is mentioned that the global PHA market size was calculated to be USD 650.66 million in 2023 and has about 9.35% CAGR from 2024 to 2030. Global Market Insights indicate that the PHA market was valued at USD 123.5 million in 2024 and is expected to grow up to USD 298.3 million (8.9% CAGR) by 2034.^95^ On the other hand, Mordor Intelligence calculated that in 2025 there will be 49.04 kilotons of PHA, which are expected to reach 141.00 kilotons by 2030, projecting a 23.52% CAGR.^96^

As the literature lacks investigations that focus on optimizing the 3D-printing parameters of PHA samples subject to dynamic mechanical analysis (DMA) testing, this research chose to enrich this topic. Dynamic Mechanical Analysis (DMA) was investigated herein, as it is an essential analytical technique for evaluating the viscoelastic properties of materials concerning time, temperature, and frequency. It provides significant insights into the mechanical behavior of polymers, composites, and other materials subjected to dynamic loads. The primary parameters measured in DMA include the storage modulus (*E′*), loss modulus (*E″*), and damping factor (tan δ), which respectively characterize material stiffness, energy dissipation, and internal friction.^97^ DMA is particularly sensitive to phase transitions, notably the glass transition temperature (*T*_g_) of polymers. In contrast to differential scanning calorimetry (DSC), DMA can detect subtle transitions and serves as an excellent tool for characterizing complex polymer systems.^98^ The frequency-dependent test assesses both elastic (solid-like) and viscous (liquid-like) responses, which is crucial for applications where materials experience cyclic or oscillatory stresses^99^ DMA provides quantitative data on a material’s stiffness and energy dissipation capabilities, which are vital for applications such as vibration damping, motor vehicle components,^100^ and aerospace materials.^97^ It facilitates online monitoring of curing reactions in thermosetting polymers and crystallization in semicrystalline polymers, which is essential for manufacturing and quality control.^101^ Overall, DMA is a highly sensitive and versatile method for understanding material behavior under dynamic loading conditions. Due to its precision in defining transitions, viscoelasticity, and mechanical properties, DMA is indispensable for material development, quality control, and failure analysis.

The thermal and rheological behaviors of the PHA material were determined using differential scanning calorimetry (DSC), and viscosity and melt flow rate (MFR) assessments, respectively. The microstructures of the specimens were evaluated using optical microscopy of their top surfaces. The storage modulus, loss modulus, and damping factor were obtained from the average and standard deviation curves during the DMA. The optimization of this research was carried out by the Taguchi design of the experiment. Four different control parameters were selected: deposition speed (*D_S_*), layer height (*L_H_*), extrusion temperature (*E*_*T*_), and extrusion width (*E_W_*). Each was set and tested at three different levels, creating a total of nine experimental runs and one additional confirmation run. The response metrics selected were the flexural storage modulus (*E* ^F′^), dynamic glass transition temperature (*DT*_g_), and damping factor at the dynamic glass transition temperature (*DFDT*_g_). Reduced quadratic regression modeling was employed, which was verified with an additional confirmation test. The efficiency of parameter optimization was revealed, judging by the derived results, while being beneficial for the research and scientific field, by beginning to investigate such topics. Through this process, the critical 3D printing affecting the response of PHA under simultaneous dynamic and thermal loading (viscoelasticity along with thermal properties) was revealed. Furthermore, the response metric values were optimized by locating the optimum values of the control parameters. Such findings contribute to the further expansion of the PHA potential, as they contribute to evaluating the 3D printed PHA parts behavior in real-life operating conditions.

## 2 Materials and Methods

### 2.1 Materials

The material employed in this work was a commercial named allPHA filament of 1.75 mm diameter, and 1,24 g/cm^3^ density, supplied by Colorfab (Belfeld, The Netherlands). In the safety datasheet, it is mentioned that it contains Titanium dioxide in an amount <2.5%. It is mentioned that it is not flammable and it does not meet the PBT and vPvB classification criteria. It does not pose an environmental hazard. It is certified by TUV Austria Belgium that it fulfills the 90% biodegradation requirements. The manufacturer states that the synthesis of ″allPHA″ is facilitated through fermentation, a natural biochemical process. By supplying bacteria with natural sugars and oils, they generate ″fat″ cells known as polyhydroxyalkanoates (PHAs). This allPHA material is anticipated to biodegrade in any environment without leaving microplastics. When bacteria are provided with natural sugars and oils, such as glucose, sucrose, vegetable oils, or fatty acids, they can produce a class of PHAs—biodegradable polyesters that serve as intracellular carbon and energy reserves. The specific type of PHA synthesized is contingent upon the carbon source and the bacterial strain utilized. From sugars such as glucose and sucrose, the resultant PHA is Polyhydroxybutyrate (PHB) or PHB-*co*-HV (a copolymer with hydroxyvalerate), classified as a short-chain-length PHA (scl-PHA). This is produced by bacteria such as Cupriavidus necator. PHB is a brittle thermoplastic, akin to polypropylene. Conversely, from oils and fatty acids, such as vegetable oil and waste cooking oil, a medium-chain-length type of PHAs (mcl-PHA) is derived. This is synthesized by bacteria such as *Pseudomonas putida* and *Pseudomonas aeruginosa*. It exhibits greater elastomeric and flexible properties than PHB, rendering it suitable for medical and packaging applications. This allPHA material is a PHA material, not a PHB one. Therefore, it should be noted that the results apply to this specific grade and can vary in other PHA grades or types. Considering the data sheet provided by the supplier, the PHA properties during 3D printing were:

- 24 MPa tensile strength
- 4.5 elongation at break
- 41 MPa flexural strength
- 3.4 kJ/m^2^ Charpy impact strength.

### 2.2 Experimental Strategy

Figure 1 presents the sequence of actions conducted in this study (left side of the figure) and the experimental design framework (right side of the figure). Figure 1a,b shows the PHA drying and its 3D printing additive manufacturing, Figure 1d,c shows the microstructural characterization and optical evaluation, respectively, while Figure 1e shows the thermomechanical analysis through DMA.

**Figure 1:**
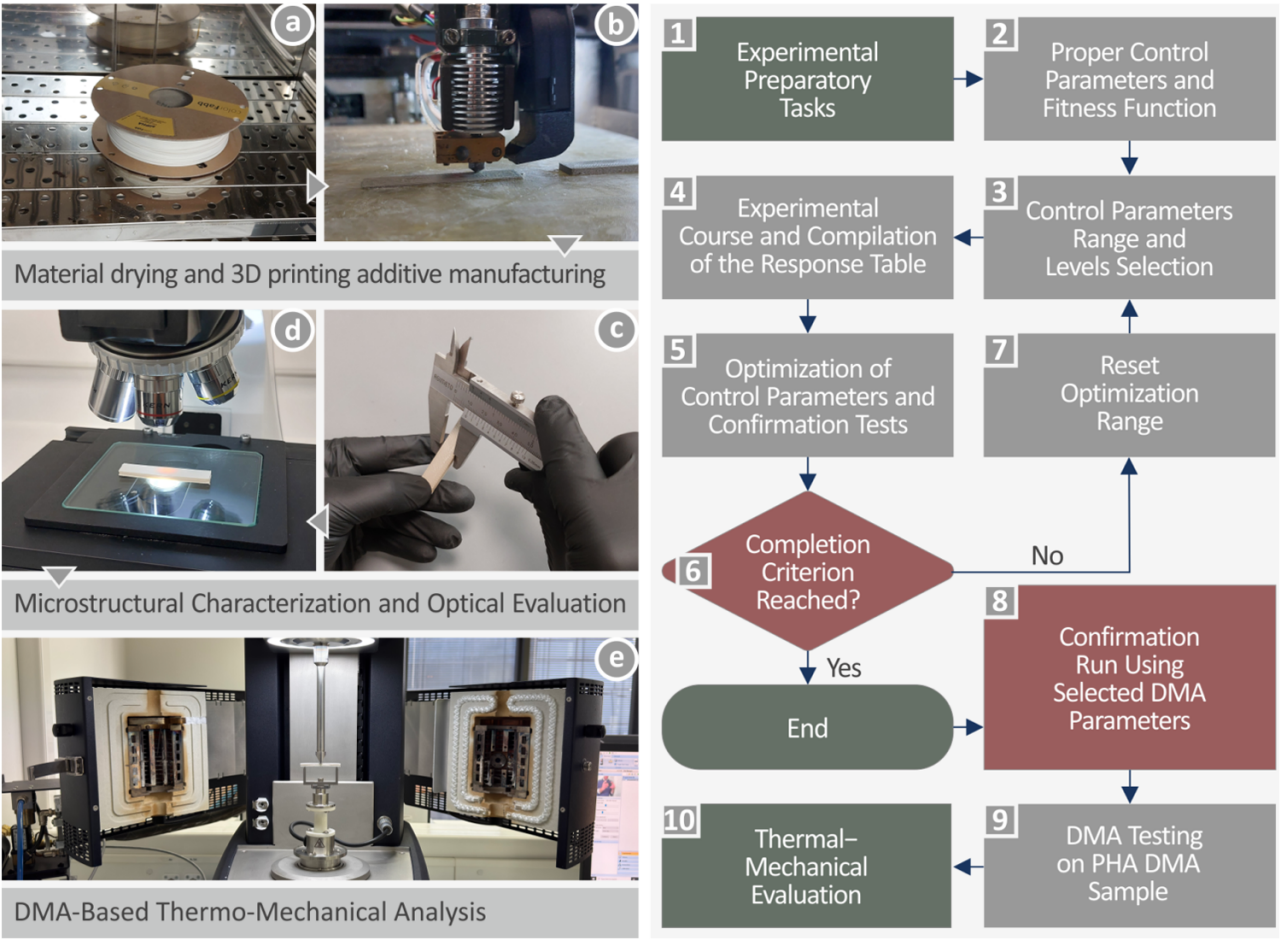
(Left side) sequence of actions during this work (a, b) material dehydration and 3D printing, (c, d) microstructural characterization and optical evaluation, (e) DMA-based thermomechanical examination and (right side) experimental design framework.

### 2.3 PHA Thermal and Rheological Examination

The thermal characteristics and rheology of the PHA material were determined by TGA, DSC, viscosity, and MFR analyses. TGA was conducted in the temperature range of 20–400 °C on a PerkinElmer Diamond apparatus (Waltham). DSC data were obtained using TA Instruments Discovery-Series DSC 25 (Delaware). Rheological evaluation was conducted using a TA Instruments DHR-20 Discovery Hybrid Rotational Rheometer (New Castle, DE (following ASTM D1238-13)). The device was accompanied by a parallel plate configuration (25 mm diameter) and an environmental test chamber, providing the ability to control the temperature. Figure 2 shows the derived information in the mass degradation graph (TGA) (Figure 2a), heat flow vs temperature graph (DSC) (Figure 2b), stress and viscosity vs shear rate graph (Figure 2b), and MFR levels for each temperature (Figure 2d). As shown from the TGA graph (Figure 2a), no degradation of the material was observed at higher extrusion temperatures. The PHA starts to acutely degrade at 280.9 °C, which is much higher than the highest extrusion temperature of 220 °C utilized in the research. From the MFR analysis, it can be observed that it begins at low temperatures and continues until higher temperatures. It should be mentioned that at 175 °C, the material did not flow, while above 190 °C, its flow was that of a liquid, and the measurement could not be taken on time. Consequently, the temperature range was selected to be between 180–190 °C, with a 2.5 °C step.

**Figure 2:**
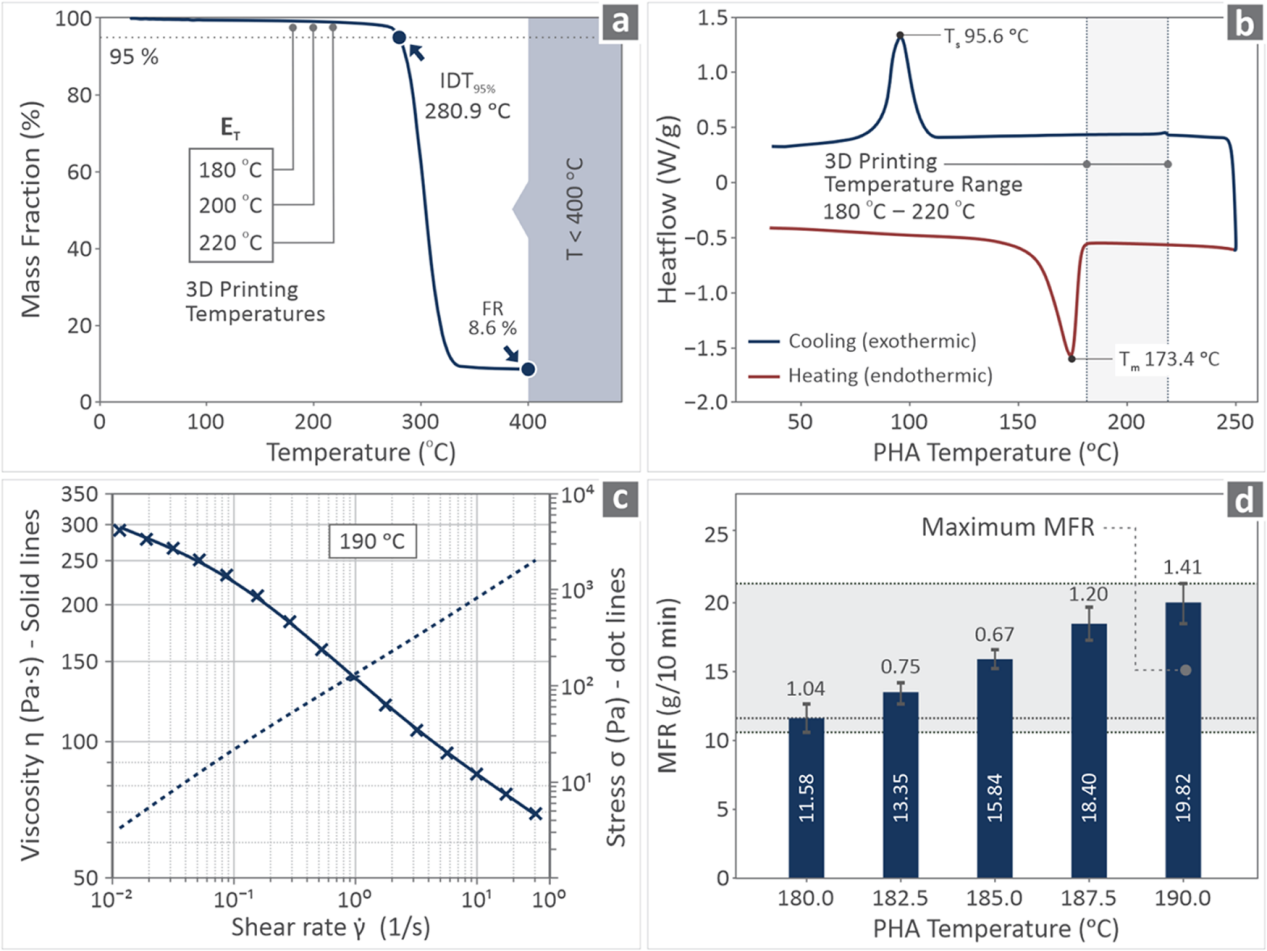
(a) TGA graph, (b) DSC graph (*T*_s_ is the *T* solid, while *T*_m_ is *T* melt), (c) stress and viscosity vs shear rate, (d) MFR levels between the temperature range of 180–190 °C (2.5 °C step).

### 2.4 PHA Coupon 3D Printing, DMA and Mechanical Testing, and Optical Evaluation

The PHA specimens were manufactured by 3D printing through MEX, in such a design as suitable for the tests that would follow. The 3D printing fixed parameters are presented in the next section in Figure 3a, along with some additional information, which will be discussed below. The apparatus utilized for the printing was a Funmat-HT 3D-Printer from Intamsys, Shanghai, China, and the geometry of the coupons was that of the 3-point bending, with dimensions of 50 × 10 × 3 mm^3^ and a 40 mm support span.

**Figure 3:**
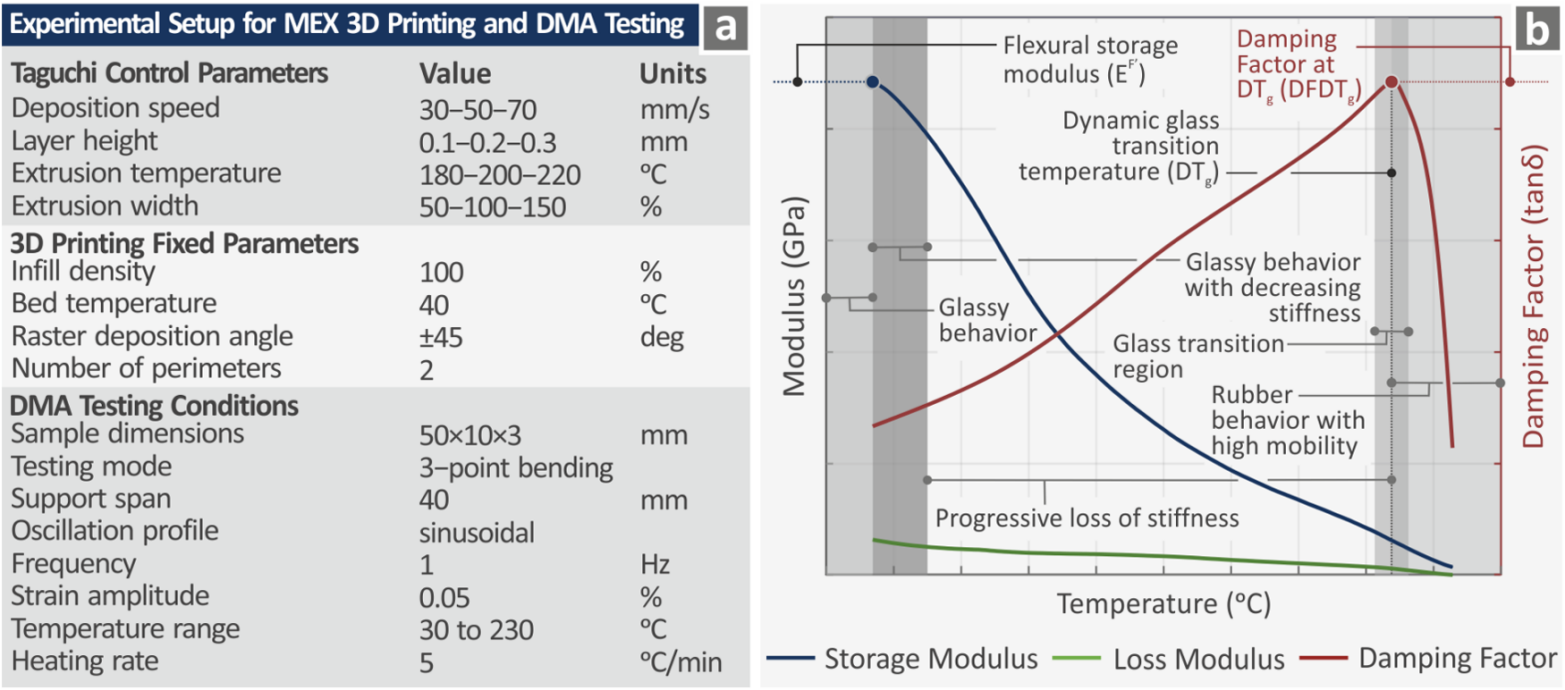
(a) Taguchi experimental parameters, the 3D printing fixed parameters, and the DMA testing conditions, (b) DMA graph components explanation.

DMA testing was conducted using a DHR 20 Discovery Hybrid Rotational Rheometer (TA Instruments, New Castle, DE) accompanied by an environmental test chamber for temperature regulation throughout the testing process. The DMA temperature range was between 30–230 °C with a 5 °C/min temperature ramp. The frequency was set at 1 Hz, while there was a strain amplitude of 0.05% and a sinusoidal oscillation profile. This information is also provided in Figure 3a and is described in the next section. All measurements were carried out in the air atmosphere.

The research focused on the rheological response, the viscoelastic behavior, and the mechanical response under thermal and dynamic loading. For completeness of the mechanical validation under static loading, tensile and impact tests were carried out. Tests were carried out following the respective standards, i.e., ASTM D638-14 for the tensile tests on an Imada MX2 apparatus, testing type V, 3.2 mm thickness samples, and ASTM D6110 on a Terco MT220 apparatus for Charpy impact tests on notched samples, respectively. Five samples were tested per case in ambient room conditions.

Optical evaluation of the samples was performed using an optical stereoscope (OZR5, 5.1 MP ODC 832 camera) from Kern, Balingen, Germany. Images from the top surfaces of the samples were captured, showing the impact of the various parameter levels on their performance and the correlation of the results.

### 2.5 Design of Experiments and Modeling Procedure

Initially, the Taguchi method was employed, with the aim of choosing cases for modeling from the orthogonal array. The purpose of its use is to avoid the complexity of classical experimental designs and the large number of simulations and experiments that would need to be conducted as the printing parameters increase.

The use of the Taguchi design of experiment provided the ability to examine the entire parameter space, while fewer experiments were necessary to reach problem solution.^102^ Normally, the orthogonal array is selected by utilizing the total degree of freedom (DOF), which is the number of factor levels minus 1, for each factor^103^ and is supposed to be lower than the DOF of the chosen orthogonal array.

Taguchi was used for the analysis due to the fact that different modeled parameters can have an impact on the outcome. The aim is to receive the most suitable parameter combination.^104^ Its proven favor^105–109^ has led to its employment in several research fields.^110^ The method provides independent parameters based on their importance for dependent objective functions,^111^ while a parameter set is created to result in the best-case and worst-case scenarios. The ANOVA (analysis of variances) statistical technique is widely utilized to interpret the experimental results derived by obtaining the contribution ratio of all parameters. It examines each parameter’s significance for the current problem in need of a solution to the problem related to DMA results. Two methods, the Linear Regression Model (LRM) and the Reduced Quadratic Regression Model (RQRM), were employed to compare their efficiency for the present analysis. The theoretical part and respective equations applied are provided in the Supporting Information.

The control parameters of the Taguchi L9 array optimization analysis herein are *D_s_* (mm/s), *L_H_* (mm), *E_T_* (°C), and *E_W_* (%), each having three levels, and are listed in Table 1 and Figure 3a. On the other hand, the response metrics were *E* ^F′^, *DT*_g_ and *DFT*_g_. The control parameters were selected according to the literature review on which parameters are the most critical in 3D printing of polymeric parts and corresponding research on 3D printed PHA parts.^18,58,112^ The parameter levels were selected based on supplier recommendations and preliminary trials. The supplier provides the optimum 3D printing settings for the PHA material which were considered for the selection of the parameter levels. Furthermore, preliminary trials were carried out and parameter levels with which processability and printability issues occurred were discarded. Through this process, the parameter levels’ range was formed. Furthermore, the thermal investigation findings and the PHA polymer datasheet, along with the respective literature were consulted.^18,58,112^ The response metrics were the ones the DMA test provides.

**Table 1:** Taguchi L9 Design: Control Parameters and Levels

**run**	***D***_***s***_ (mm/s)	***L***_***H***_ **(mm)**	***E***_***T***_ **(°C)**	***E***_***W***_ **(%)**
1	30	0.1	180	50
2	30	0.2	200	100
3	30	0.3	220	150
4	50	0.1	200	150
5	50	0.2	220	50
6	50	0.3	180	100
7	70	0.1	220	100
8	70	0.2	180	150
9	70	0.3	200	50

In Figure 3a, the Taguchi experimental parameters, the 3D printing fixed parameters, and the DMA testing conditions are presented. Figure 3b shows a typical example of DMA graph components (thus the lack of numbers in the axes), to depict the various zones, namely those with glassy behavior, glassy behavior with increasing stiffness, progressive loss stiffness, glass transition region, and rubber behavior with high mobility.^98^ In addition, the three response metrics of this research work have been marked on the graphs for comprehensiveness: *E* ^F′^, *DT*_g_ and *DFDT*_g_. *E* ^F′^ is the first value of the storage modulus curve, *DT*_g_ is the peak of the damping factor, and *DFDT*_g_ is the damping factor at *DT*_g_. The characteristic measures and metrics responses are highlighted in this graph (Figure 3b).

## 3 Results

### 3.1 Mechanical Testing, DMA Analysis, and Optical Microscopy Results

The experimental results from the static loading tests are presented in the following Table 2.

**Table 2:** Static Loading Response of the 3D-Printed PHA Samples

tensile strength (MPa)	Young’s modulus (MPa)	Charpy impact strength (kJ/m^2^)
19.8 ± 0.7	108.2 ± 13.7	21.4 ± 1.4

The results derived from DMA analysis and optical microscopy are presented in the following figures and tables. In Figure 4a–i there are the DMA graphs belonging to the nine runs of this investigation, respectively, showing the storage modulus, loss modulus, and damping factor curves, as well as the control parameter values representing each run. The bolt curves represent the average value, while the wider colored areas are the standard deviations. Different responses can be observed among the runs, especially for the damping factor parameter. For the modulus parameter, the deviations are low and in most cases were found at lower temperatures. On the other hand, for the damping factor, there are runs (run 1, 8) with low deviations, runs with higher deviations in midrange temperatures (run 2, 3, 5), and runs with higher deviations in higher temperatures (run 4, 6, 7, 9). The damping factor (tan δ) is defined as the ratio of the viscous (loss) modulus to the elastic (storage) modulus. It quantifies the energy dissipation of a material during deformation and its internal friction. Deviations in tan δ values between samples can be attributed to the different print parameters, which lead to differences in layer adhesion. Furthermore, microstructural defects can occur at specific parameter sets.

**Figure 4:**
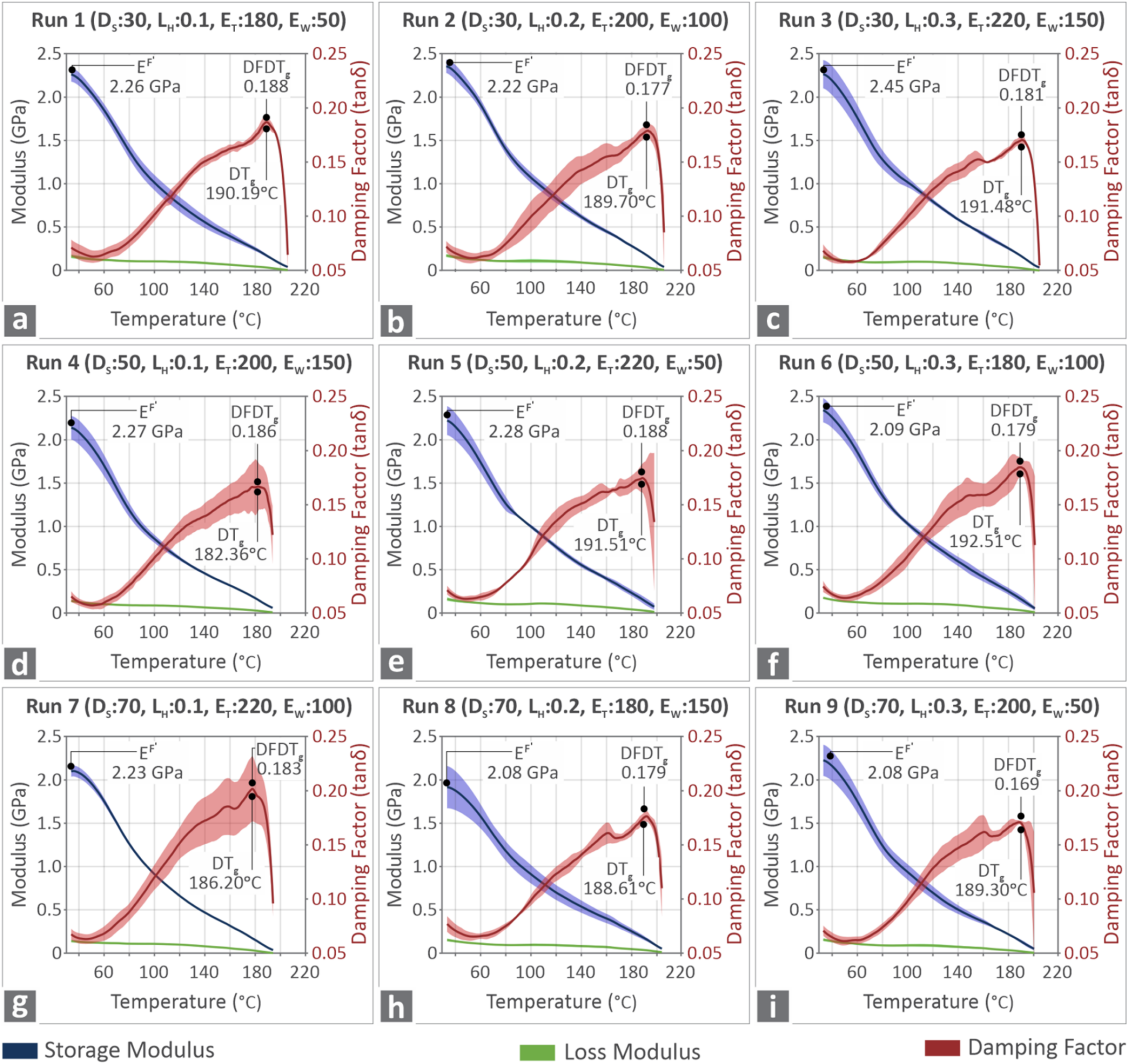
DMA curves of the nine runs, showing the storage and loss modulus, as well as the damping factor, and indicating the average and standard deviation (color zones) (a–i) Run 1 to Run 9 samples, respectively.

In Figure 5, images obtained from optical microscopy conducted on the nine runs of this work are shown. The top surface of each sample was imaged at 10× magnification. The printing parameter values of all runs are also depicted. In some cases, such as in Run 9 (Figure 5i), the layering could be characterized as thinner or less uniformly distributed. This could be due to the inability to fill properly owing to the high deposition speed.

**Figure 5:**
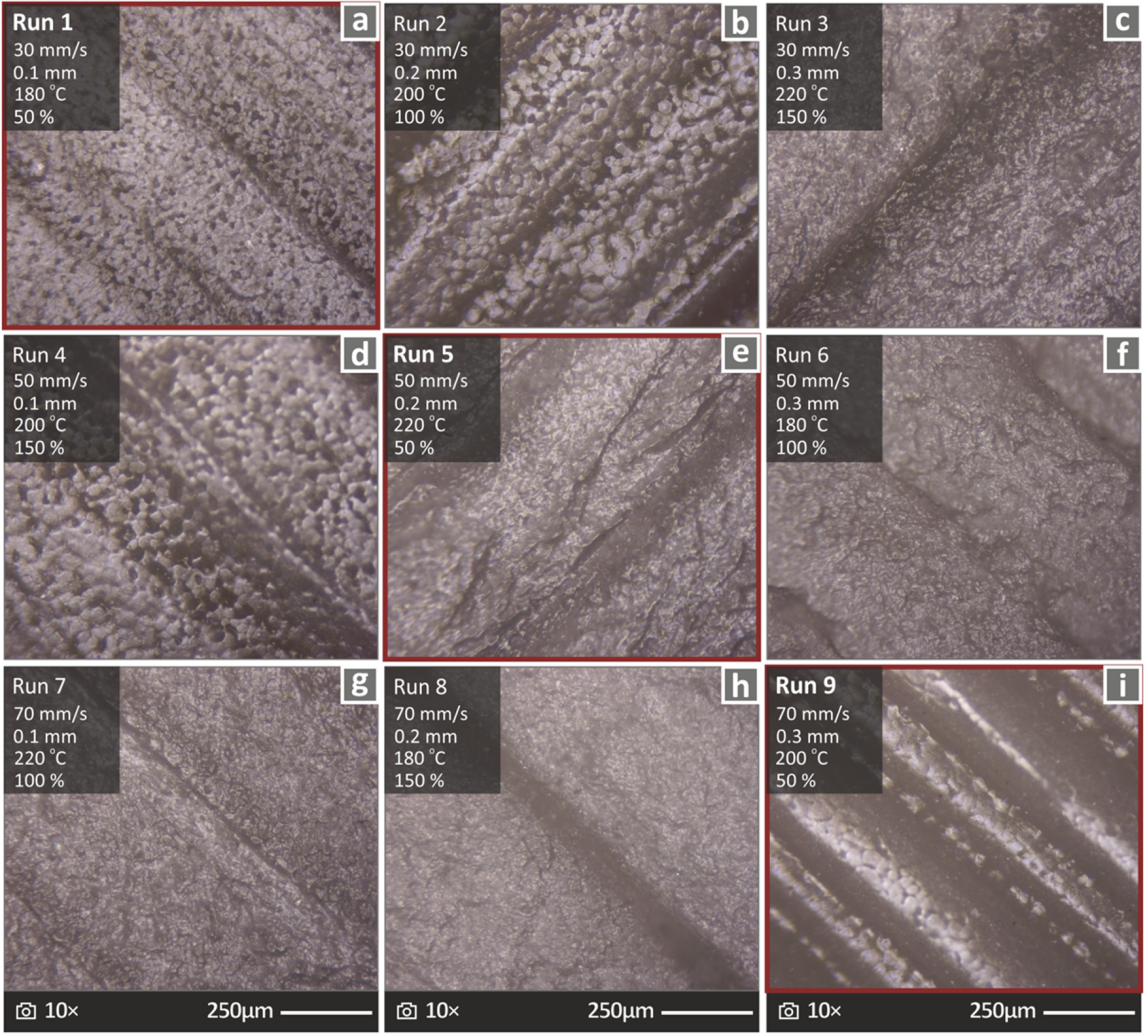
Images captured in 10× magnification, during optical microscopy conduction, on the samples belonging to the nine runs, and the respective control parameter values (a–i) Run 1 to Run 9 samples respectively.

Table 3 shows the control parameter rankings for means *E* ^F′^, *DT*_g_, *DFDT*_g_, and Table 4 shows the average and standard deviations of the measured responses for *E* ^F′^,’ *DT*_g_, *DFDT*_g_. Figure 6 shows the main effect plots and the ranks of the three response metrics, namely *E* ^F′^ (Figure 6a), *DT*_g_ (Figure 6b), and *DFDT*_g_ (Figure 6c), as a function of the four printing parameters. In the case of *E* ^F′^, *D*_S_ seems to be the most influential control parameter, as it is placed in Rank 1, while the least influential control parameter (in Rank 4) seems to be *L*_H_. For *DT*_g_ and *DFDT*_g_, *L*_H_ is placed in Rank 1, whereas Rank 4 is occupied by *D*_S_ and *E*_W_. Additional information can be found in the supplementary work of this study, where Table S1 provides the measured *E* ^F′^, *DT*_*g*_, *DFDT*_g_ for each experimental run and five replicas per run.

**Figure 6:**
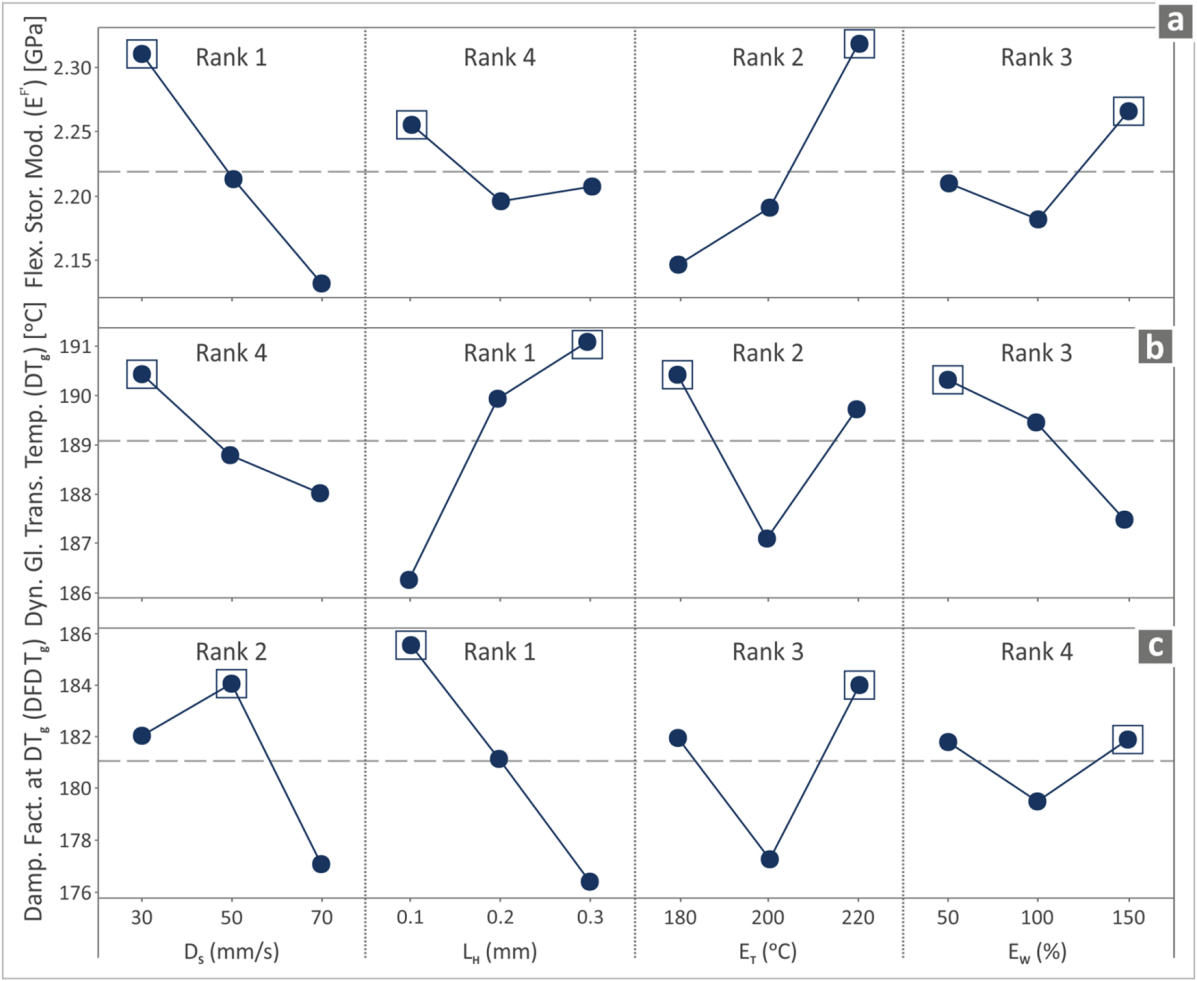
Main effect plots and ranks considering (a) *E* ^F′^, (b) *DT*_g_ and (c) *DFDT*_g_ as a function of *D_S_*, *L*_H_, *E_T_*, and *E_W_*.

**Table 3:** Control Parameters Ranking for Means *E* ^F′^, *DT*_g_, *DFDT*_g_

**level**	***D***_***s***_(mm/s)	***L***_***H***_ **(mm)**	***E***_***T***_ **(°C)**	***E***_***W***_ **(%)**
***E***^***F*** ′^(**MPa**)				
**1**	2311	2255	2146	2210
**2**	2214	2196	2191	2182
**3**	2132	2206	2320	2265
**delta**	179	58	175	83
**rank**	1	4	2	3
***DT***_***g***_ **(°C)**				
**1**	190.5	186.2	190.4	190.3
**2**	188.8	189.9	187.1	189.5
**3**	188	191.1	189.7	187.5
**delta**	2.4	4.8	3.3	2.8
**rank**	4	1	2	3
***DFDT***_**g**_				
**1**	0.1821	0.1857	0.182	0.1818
**2**	0.1841	0.1812	0.1773	0.1795
**3**	0.1771	0.1764	0.184	0.1819
**delta**	0.007	0.0092	0.0067	0.0024
**rank**	2	1	3	4

**Table 4:** Average and Standard Deviation Values of Measured Responses for *E* ^*F*′^, *DT*_g_, *DFDT*_g_

**run**	***E***^***F*** ′^ (**MPa**)	***DT***_**g**_ **(°** ***C*** **)**	***DFDT***_**g**_
1	2264.0 ± 65.4	190.19 ± 2.60	0.188 ± 0.006
2	2223.7 ± 52.1	189.70 ± 1.80	0.177 ± 0.006
3	2446.1 ± 11.8	191.48 ± 1.60	0.181 ± 0.002
4	2267.7 ± 74.1	182.36 ± 2.44	0.186 ± 0.013
5	2282.7 ± 57.9	191.51 ± 3.09	0.188 ± 0.005
6	2090.5 ± 107.5	192.51 ± 5.65	0.179 ± 0.003
7	2232.2 ± 118.4	186.20 ± 7.43	0.183 ± 0.008
8	2082.1 ± 76.0	188.61 ± 2.43	0.179 ± 0.007
9	2082.1 ± 158.1	189.30 ± 2.04	0.169 ± 0.002

Tables 5–7 include the polynomial ANOVA information for *E* ^F′^, *DT*_g_, *DFDT*_g_, respectively, versus the *D_S_*, *L_H_*, *E_T_*, *E_W_* control parameters. Each table is accompanied by the respective equations for the current response metric considering the Reduced Quadratic Regression Model. Table 5 and eq 1 are about *E* ^F′^, Table 6 and eq 2 are about *DT*_g_, and Table 7 and eq 3 are about *DFDT*_g_. The *R* values are observed to be slightly over 70%, below 50%, or slightly below 52%.

**Table 5:** Polynomial ANOVA, RQRM, *E* ^F′^ vs *D_S_*, *L_H_*, *E_T_*, *E_W_*

source	*DF*	adj *SS*	adj *MS*	*F*-value	*P*-value
regression	8	342,624	42,827.9	5.33	0.002
*D*_*S*_	1	3994	3993.9	0.50	0.490
*L*_*H*_	1	9550	9549.9	1.19	0.290
*E*_*T*_	1	8384	8383.6	1.04	0.321
*E*_*W*_	1	13,756	13,755.8	1.71	0.207
*D*_*S*_^2^	1	390	390.1	0.05	0.828
*L*_*H*_^2^	1	7041	7040.9	0.88	0.362
*E*_*T*_^2^	1	10,466	10,466.4	1.30	0.269
*E*_*W*_^2^	1	18,373	18,372.5	2.29	0.148
error	18	144,670	8037.2		
total	26				
*R* ^2^	70.31%				
*R*^2^ (adj)	57.12%				
*R*^2^ (pred)	33.20%				

**Table 6:** Polynomial ANOVA, RQRM, *DT*_*g*_ vs *D*_*S*_, *L*_*H*_, *E*_*T*_, *E*_*W*_

source	*DF*	adj *SS*	adj *MS*	*F*-value	*P*-value
regression	8	236.144	29.5181	2.12	0.088
*D*_*S*_	1	2.837	2.8372	0.20	0.657
*L*_*H*_	1	20.599	20.5990	1.48	0.239
*E*_*T*_	1	53.176	53.1755	3.82	0.066
*E*_*W*_	1	0.243	0.2427	0.02	0.896
*D*_*S*_^2^			1.2180	0.09	0.771
	1	9.618	9.6182	0.69	0.417
*E*_*T*_^2^	1	52.589	52.5893	3.78	0.068
*E*_*W*_^2^	1	1.878	1.8779	0.13	0.718
error	18	250.496	13.9164		
total	26				
*R* ^2^	48.53%				
*R*^2^ (adj)	25.65%				
*R*^2^ (pred)	0.00%				

**Table 7:** Polynomial ANOVA, RQRM, *DFDT*_*g*_ vs *D*_*S*_, *L*_*H*_, *E*_*T*_, *E*_*W*_

source	*DF*	adj *SS*	adj *MS*	*F*-value	*P*-value
regression	8	0.000861	0.000108	2.40	0.059
*D*_*S*_	1	0.000094	0.000094	2.10	0.165
*L*_*H*_	1	0.000007	0.000007	0.15	0.707
*E*_*T*_	1	0.000191	0.000191	4.26	0.054
*E*_*W*_	1	0.000032	0.000032	0.71	0.412
*D*_S_^2^	1	0.000120	0.000120	2.69	0.119
*L*_*H*_^2^	1	0.000000	0.000000	0.00	0.971
*E*_*T*_^2^	1	0.000194	0.000194	4.34	0.052
*E*_*W*_^2^	1	0.000033	0.000033	0.73	0.405
error	18	0.000806	0.000045		
total	26				
*R* ^2^	51.62%				
*R*^2^ (adj)	30.12%				
*R*^2^ (pred)	0.00%

1 $$E^{F\prime }=6054-6.49\times D_{S}-1612\times L_{H}-37.4\times E_{T}-3.87\times E_{W}+0.0202\times D_{S}^{2}+3426\times L_{H}^{2}+0.1044\times E_{T}^{2}+0.0221\times E_{W}^{2}$$

2 $$DT_{g}=484-0.173\times D_{S}+74.9\times L_{H}-2.98\times E_{T}+0.016\times E_{W}+0.00113\times D_{S}^{2}-127\times L_{H}^{2}+0.00740\times E_{T}^{2}-0.000224\times E_{W}^{2}$$

3 $$\mathrm{DFD}T_{g}=0.734+0.000996\times D_{S}-0.042\times L_{H}-0.00564\times E_{T}-0.000186\times E_{W}-0.000011\times D_{S}^{2}-0.010\times L_{H}^{2}+0.000014\times E_{T}^{2}+0.000001\times E_{W}^{2}$$

A comparison of the LRM and RQRM models is presented in Table 8. By observing the *R* values, it is clear that the RQRM model provided higher levels in all cases, which explains how it was selected over the LRM. The LRM equations for the three response metrics are presented in eqs 4–6. Moreover, the measured *E* ^F′^, *DT*_*g*_, *DFDT*_g_ for the five replicas of the confirmation experimental run can be found in the Supporting Information.

**Table 8:** Comparison between LRM and RQRM

	LRM			RQRM		
	*R* ^2^	*R*^2^ (adj)	*F*-value	*R* ^2^	*R*^2^ (adj)	*F*-value
*EF*′ (MPa)	62.87%	56.12%	9.31	70.31%	57.12%	5.33
*DT*_g_ (°C)	35.11%	23.31%	2.98	48.53%	25.65%	2.12
*DFDT*_g_	30.79%	18.21%	2.45	51.62%	30.12%	2.40

**Linear Regression Model Equations for** *E* ^*F*′^, *DT*_g_, *DFDT*_g_

4 $$E^{F\prime }=1562-4.48\times D_{S}-242\times L_{H}+4.37\times E_{T}+0.557\times E_{W}$$

5 $$DT_{g}=193.66-0.0604\times D_{S}+24.24\times L_{H}-0.0177\times E_{T}-0.0285\times E_{W}$$

6 $$\mathrm{DFD}T_{g}=0.1866-0.000124\times D_{S}-0.0462\times L_{H}+0.000050\times E_{T}+0.000001\times E_{W}$$

To evaluate the predictive accuracy of the regression models, residual analysis was carried out and the respective graphs are provided in the Supporting Information (Figures S3–S5). In the three supplementary figures standard residual diagnostics, normal probability plot of residuals, residuals versus fits, histogram of residuals, and residuals versus order, for each of the three predictive variables, are provided. These plots support the validity of the model assumptions, including normality, homoscedasticity, and independence of residuals. The predictive performance of the models was clarified using leave-one-out cross-validation. For *E* ^F′^, the model shows reasonable predictive accuracy, with *R*_pred_^2^ reaching 33%. In contrast, the models for *DT*_g_ and *DFDT*_g_ yield near-zero *R*_pred_^2^ values, indicating that they are not reliable for prediction. These models are more useful for highlighting the factors that matter the most and locating general patterns in the process.

Run 10 was the confirmation run utilized herein. The respective control parameters are presented in Table 9. Then, in Table 10, the average and standard deviation values of the three response metrics for confirmation Run 10. In addition, Table 11 is a validation table for Run 10 with the actual values of *E* ^F′^, *DT*_g_ and *DFDT*_g_, the predicted values, and the error occurring between them, which seems to be minimal to negligible.

**Table 9:** Control Parameters for the Confirmation Run

**run**	***D***_***s***_ (mm/s)	***L***_***H***_ **(mm)**	***E**_**T**_* **(°C)**	***E***_***W***_ **(** ***%*** **)**
10	30	0.30	220	100

**Table 10:** Average and Standard Deviation of the Responses for *E* ^*F*′^, *DT*_g_, *DFDT*_g_, for the Confirmation Run

**run**	***E*** ^**F′**^ **(MPa)**	***DT***_**g**_ **(°C)**	***DFDT***_**g**_
10	2345.7 ± 80.1	191.23 ± 3.43	0.172 ± 0.004

**Table 11:** Validation Table

**run**		**10**
actual	*E*^F′^ (MPa)	2345.68
	*DT*_g_ (°C)	191.23
	*DFDT*_g_	0.17
predicted	*E*^F′^ (MPa)	2361.18
	*DT*_g_ (°C)	192.79
	*DFDT*_g_	0.17
absolute error	*E*^F′^ (%)	0.7
	*DT*_g_ (%)	0.8
	*DFDT*_g_ (%)	1.8

Figure 7 shows the experimental and calculated values for the three response metrics in graphs, namely *E* ^F’^ (Figure 7a), *E* ^F′^, *DT*_g_ (Figure 7b), *DT*_g_ and *DFDT*_g_, (Figure 7c) *DFDT*_g_. The blue and red dots represent the experimental and calculated values, respectively, placed in increasing order, showing their deviation. For each response metric, the MAPE (mean absolute percentage error) and Durbin-Watson parameters were calculated and depicted in the graph. For the MAPE metric, values lower than 10% depict that the prediction was highly accurate.^113,114^ Herein, the values were much lower than 10% in the range between 1 and 3%, showing the prediction accuracy. The Durbin-Watson (DW) test is utilized to assess the autocorrelation of residuals within a statistical model or regression analysis. The DW statistic ranges from 0 to 4, with a value of 2.0 indicating the absence of autocorrelation in the sample. Values between 0 and less than 2 denote positive autocorrelation, whereas values from 2 to 4 indicate negative autocorrelation. Herein, for all response metrics, values lower than 2 were calculated.^115^ Positive autocorrelation refers to the tendency of values to be similar to their preceding ones, indicating persistence in the values. Additional information is provided in the Supporting Information for this research in Figures S1 and S2, which present the box plots and surface 3D plots.

**Figure 7:**
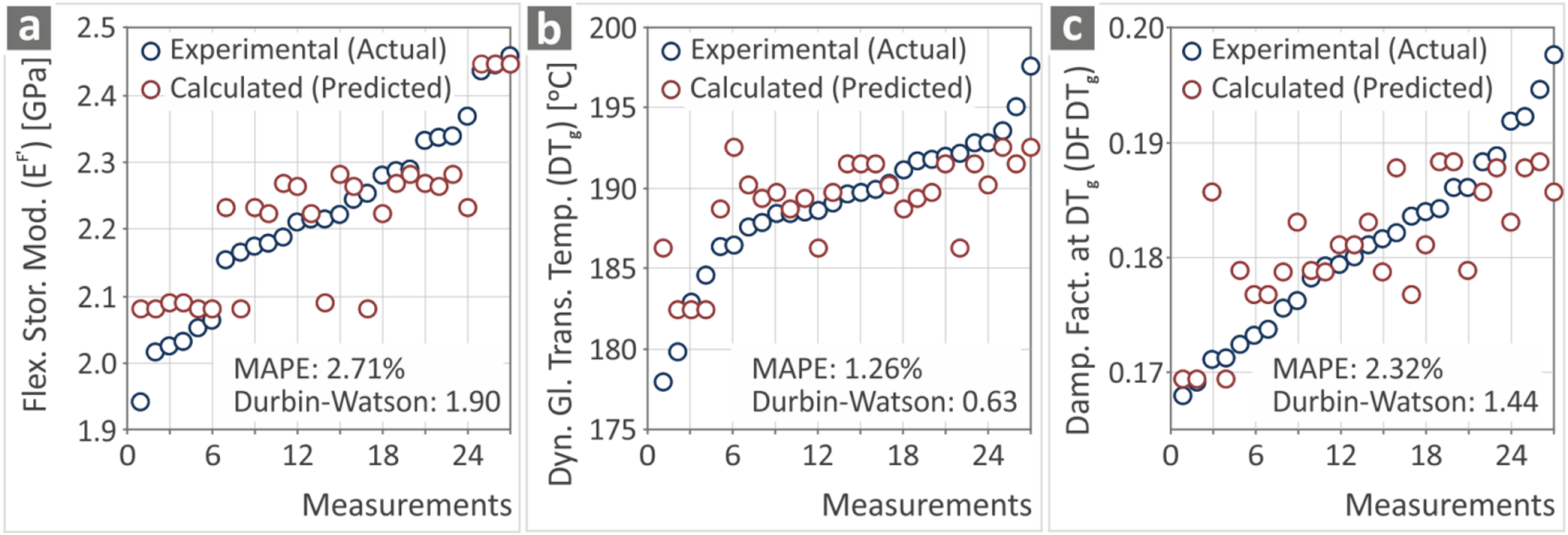
Graphs of experimental and calculated values of (a) *E* ^F′^, (b) *DT*_g_ and (c) *DFDT*_g_.

## 4 Discussion

The aim herein was to reveal the rheology and the critical 3D printing settings affecting the thermomechanical (viscoelastic) response of 3D printed parts made with the sustainable nature-sourced PHA biopolymer. Melt Flow Rate (MFR) is a pivotal parameter in assessing the processability and flow behavior of polymers in extrusion-based 3D printing. In polyhydroxyalkanoate (PHA) polymers, MFR typically increases with temperature due to changes in viscosity and molecular mobility. This was verified herein, through the MFR measurements (Figure 2c). PHA use in 3D printing is attributed to its environmental benefits and satisfactory mechanical performance.^116–118^ Melt Flow Rate (MFR), defined as the mass of polymer flowing through a standard capillary under a specified load within 10 min, serves as a crucial indicator of the melt viscosity and processability of the polymer in extrusion.^116^ The MFR of PHA is highly sensitive to temperature variations. As temperature rises, polymer chains gain kinetic energy, diminishing intermolecular interactions and consequently reducing melt viscosity.^119,120^ This transition facilitates the free flow of the polymer, which is essential for achieving smooth extrusion and layer adhesion in 3D printing.^121^ The primary factor contributing to the increase in MFR with temperature is the reduction in melt viscosity. Elevated temperatures disrupt van der Waals forces and secondary bonds within the polymer matrix, thereby enhancing chain mobility.^122^ The decreased resistance to flow allows for higher throughput during extrusion, which is advantageous for maintaining stable print quality.^123,124^ High temperatures also promote molecular mobility and chain relaxation, enabling PHA to flow through the nozzle with reduced shear stress. This is crucial in preventing nozzle clogging and ensuring consistent layer deposition.^125,126^ While an increased MFR can enhance print speed and layer adhesion, excessively high temperatures may lead to material degradation or excessive sagging during deposition.^4^ Therefore, optimal temperature settings are necessary to balance flowability and mechanical strength.^64^ The temperatures applied to the material during the extrusion process do not cause any material degradation. Overall, the increase in MFR with temperature in 3D-printed PHA polymers is primarily driven by reductions in viscosity and enhanced molecular mobility.

Furthermore, the DMA-based analysis provided the respective curves of the storage modulus, loss modulus, and damping factors for PHA 3D printed examples. An L9 orthogonal array was formed during the experimental design process with four control parameters (3D printing settings). Parameters such as infill density were omitted due to their known detrimental effects on the mechanical properties of 3D-printed components. The selected print orientation is a standard configuration commonly employed in research and 3D printing to mitigate anisotropy. Nozzle diameter was not included in the analysis, as an L9 array was established, as mentioned, and incorporating additional parameters would substantially increase the experimental workload. Since DMA testing is extremely time-consuming, both in setup and execution, using a larger experimental design, such as L16 or L25 would have led to the need for significantly more experiments, which was not realistic within the context of a single study. With the L9 array formed herein, the key effects were evaluated in a feasible experimental scenario. The parameters chosen for evaluation are essential, as they significantly affect the samples’ response, as demonstrated by the research findings. Not considering additional parameters can be stated as a limitation of the current research.

The average and standard deviation for each point in the nine runs were calculated and revealed (Figure 4, Table 4). The flexural storage modulus is a pivotal parameter for evaluating the stiffness and load-bearing capacity of 3D-printed polyhydroxyalkanoate (PHA) polymers.^116^ It quantifies the material’s capacity to elastically store energy under bending stresses, which is essential for assessing mechanical performance in structural and biomedical applications. This modulus is vital for load-bearing applications and maintaining dimensional stability under mechanical stress.^119,120^ A high flexural storage modulus indicates strong resistance to bending and deformation under load, which is critical for load-bearing structural components printed with PHA.^122^ Increased flexural stiffness enhances the material’s suitability for load-bearing applications, such as orthopedic implants and mechanical parts.^123,124^ Herein, the optimization process achieved a ∼ 15% increase in this metric (Run 3 vs Run 8), indicating how the proper selection of parameters can improve the PHA parts’ mechanical performance. PHA’s ability to maintain its flexural properties under cyclic loading is crucial for applications subjected to repeated mechanical stress. Fatigue resistance, enhanced by characterizing the flexural storage modulus, extends the long-term durability of printed parts.^4^ Understanding the flexural storage modulus allows for the optimization of printing parameters to achieve higher stiffness and load distribution. This is vital in medical devices, automotive components, and structural frames where mechanical reliability is paramount.^64^

The dynamic glass transition temperature (*T*_g_) is a critical thermal property that significantly influences the mechanical performance and thermal stability of 3D-printed polyhydroxyalkanoate (PHA) polymers. Tg denotes the temperature range at which the polymer transitions from a rigid, glassy state to a more pliable, rubbery state, thereby affecting material behavior during the printing process and in the final product applications. It also governs the transition between its brittle and ductile behavior.^116^ Determining the dynamic *T*_g_ of PHA is crucial for ensuring structural integrity under thermal loading conditions.^119,120^ The dynamic *T*_g_ is influenced by polymer structure, crystallinity, and processing conditions, and it determines the material’s capacity to withstand mechanical deformation at elevated temperatures. Precise regulation of dynamic *T*_g_ ensures that 3D-printed PHA components exhibit optimal mechanical properties, such as toughness and impact resistance.^123,124^ Herein, the optimization achieved a 9 °C increase in this metric (∼5% increase, Run 3 vs Run 4). This might seem like a small increase, but still, it can be proven vital in real-life applications.

The damping factor (tan δ) at the dynamic glass transition temperature (*T*_g_), defined as the loss modulus/storage modulus, is a pivotal parameter in characterizing the viscoelastic properties of 3D-printed polyhydroxyalkanoate (PHA) polymers. This parameter elucidates the material’s capacity for energy dissipation, influences mechanical stability, and determines interlayer adhesion in 3D printing. Understanding the viscoelastic behavior, particularly the damping factor (tan δ) at the dynamic glass transition temperature (*T*_g_), is essential for optimizing performance in most applications.^116^ This property is especially critical in 3D-printed components, where interlayer adhesion, thermal stability, and impact resistance are key considerations.^119,120^ A higher tan­(δ) at *T*_g_ signifies greater energy absorption, enhancing the material’s toughness and impact strength,^122^ through enhanced molecular mobility and adhesion between printed layers, mitigating common issues such as delamination and poor interfaces.^123,124^ A strong damping capacity at Tg allows materials to effectively dissipate vibrational energy, which is highly beneficial in noise-suppression and mechanical damping applications.^125,126^ This characteristic is of great importance for 3D-printed components in dynamic mechanical simulations. Herein, optimization achieved a ∼10% increase for this metric (Run 1 vs Run 9).

The main effect plots that were created indicated the significant impact of *L_H_* on both *DT*_g_ and *DFDT*_g_. However, as *L_H_* increased, *DT*_g_ increased, whereas *DFDT*_g_ decreased. Moreover, *D_S_* was a control parameter that significantly influenced importantly the *E* ^F′^, causing its decrease while increasing. Therefore, from the analysis, these two were revealed as the most critical parameters. Surprisingly, the nozzle temperature was not found to be a critical parameter. Still, high values of this parameter significantly improve the flexural storage modulus, and it is ranked as no 2 in importance for this metric. Strand width was not found to be a critical parameter for the response metrics of this research.

Two regression models were then applied. The analysis showed that the LRM model achieved rather low scores in R values, and it can be assumed that it fails to model the hypothesis. The RQRM achieved much better scores, still, the R values achieved, related to the prediction accuracy were not exceptional (48.53–70.31%). This might mean in theory that the regression model did not capture the underlying pattern in the data well, due to more complex relations between the parameters. Furthermore, low *F* and *P* values were found in most of the metrics in RQRM. The *F*-value is employed to evaluate the null hypothesis that none of the independent variables exert a statistically significant effect on the dependent variable. In this context, if the *F*-values are small, it is indicated that the independent variables account for a minimal portion of the variance in the dependent variable. The *P*-value corresponds to the significance level of the *F*-test. A low *P*-value consistently indicates statistical significance, suggesting that at least one independent variable influences the model. However, if the *F*-values are small, the effect size or the variance explained by the model is negligible, despite statistical significance. This may imply in theory that the model is statistically significant, but the effect is not substantial.

However, for the *DT*_g_ and *DFDT*_g_ response metrics, the *R* ^2^ (pred) values are close to 0%, suggesting poor model generalizability for these metrics. In the context of ANOVA, the coefficient of determination (*R* ^2^) functions as a metric of merit, reflecting the proportion of variance in the dependent variable that is accounted for by the independent variable(s) within the model. An *R* ^2^ value approaching zero indicates that the model explains only a minimal portion of the variation in the response variable. This result suggests that the independent variable(s) contribute negligibly, if at all, to predicting and elucidating the outcome, leaving the majority of the variability unexplained. Such a finding implies that the treatment factor or grouping variable is not significantly correlated with the dependent variable, at least within the scope of the data and model under examination. From a model-evaluation perspective, an R^2^ estimate near zero diminishes the model’s practical relevance, indicating that it offers limited insight into the structure or dynamics of the data-generating process. It may also suggest the necessity to incorporate additional explanatory variables, refine the model’s functional form, or consider whether other sources of variability—such as interaction effects, covariates, or latent variables—are influencing the response. In summary, an *R* ^2^ value close to zero in a high ANOVA *R* ^2^ context signifies a model’s inadequacy in explanation, thereby reducing the validity of any outcomes interpreted concerning the independent and dependent variable relationship.

Furthermore, many *P*-values in the ANOVA tables are >0.05 (a high *P*-value), in combination with low *F*-values. Low *F*-values coupled with high *P*-values bear significant implications for the validity of the model’s interpretation. A low *F*-value indicates that the variation in the response variable attributable to the independent variable(s) is minimal compared to the inherent variability within the groups. Conversely, a high *P*-value—typically considered as one exceeding the conventional significance level (e.g., α = 0.05)—suggests a failure to reject the null hypothesis, which posits that all group means are equal. This outcome signifies that the differences between group means are not statistically significant and can be attributed to random fluctuations rather than a genuine effect of the explanatory variable(s). Collectively, these statistical tests imply that the model possesses limited explanatory power and is insufficient to provide conclusive evidence of systematic group differences for the specific metrics these values were derived. Such findings may necessitate remodeling the structure, incorporating additional or alternative predictor variables, or employing advanced modeling techniques to account for latent sources of variation or nonlinear effects.

To evaluate the prediction accuracy of the equations produced during regression, a confirmation run was performed. The experimental findings were compared with the predicted values. Despite the low *R* values, the deviation between predicted and experimental values in the confirmation run was negligible (less than 2% deviation). This shows that the prediction models can be considered reliable for use, at least within the control parameter values range examined. The contrast between the low *R* ^2^ and the close match in the confirmation run was evaluated as a coincidence.

## 5 Conclusions

The purpose of this research was to fabricate PHA 3D printed specimens for DMA testing in 3-point bending and investigate their thermomechanical behavior. After the 3D printing process, the microstructure of the specimens was characterized, and optical evaluation was performed before the DMA-based thermomechanical analysis. The experimental design was Taguchi L9, featuring four tunable 3D printing factors (deposition speed, layer height, extrusion temperature, and extrusion width) and three measured thermomechanical properties (*E* ^F′^, *DT*_g_ and *DFDT*_g_).

DSC, viscosity, and MFR analyses were conducted to characterize the thermal effects of PHA. The research provided information regarding the 3D printing temperature range, PHA melting temperature, and temperature range where the material was neither fluid nor unable to melt. The microstructure of the specimens was also examined through optical microscopy, revealing any detected failures, considering layering, material distribution, and sample quality in general. There were samples whose material distribution seemed to be affected by the deposition speed, judging by their inability to be evenly filled.

Through the analysis, the impact of each control parameter on the three responses was revealed; especially the *D_S_* and *L_H_* parameters were the most critical ones. The material deposition, as well as the quality of the samples’ 3D printing, was captured by optical microscopy and displayed in the respective images. Regression led to the formation of prediction models, with excellent accuracy (less than 2% deviation between the experimental and the predicted values). Furthermore, optimization managed to improve the response metric values (*E* ^F′^ was improved by ∼15%). Overall, the investigation revealed the viscoelastic and thermomechanical behavior of 3D PHA samples. Such information is vital for the design of parts, thus, the merit of the provided findings, enriching the existing literature. Future research can broaden the range of the 3D printed settings examined, and examine additional parameters and response metrics, as the provided findings are not reliable for use outside the examined range.

## Supplementary Material



## Data Availability

The raw/processed data required to reproduce these findings cannot be shared because of technical or time limitations.

## Notes

Design of experiments and modeling procedure (S1), experimental data (S2), measured *E* ^F′^, *DT*_g_, *DFT*_g_ for each experimental run and five replicas per run (Table S1), measured *E* ^F′^, *DT*_g_, for the five replicas of the confirmation experimental run (Table S2), box plots and ranks, considering (a) *E* ^F′^, (b) *DT*_g_, and (c) *DFT*_g_ (Figure S1); surface 3D plots showing (a–c) Rank 1 and 2 and (d–f) Rank 3 and 4 for each response (Figure S2), residual plots for the flexural storage modulus (Figure S3), residual plots for the glass transition temperature (Figure S4), and residual plots for the damping factor (Figure S5) (PDF)
