# Supplementary material for: The Impact of MEX 3D Printing Key Control Settings on the Rheology and DMA Response of Bacteria-Derived PHA
Source: ACS Omega. 2025 Jun 23;10(25):27632–47. doi: 10.1021/acsomega.5c04386 (PMC12224097; doi:10.1021/acsomega.5c04386)
Supplement: Supplementary file 1 [file ao5c04386_si_001.pdf]

Supplementary for the article:

**The impact of MEX 3D printing key control settings on the rheology and DMA response of bacteria-derived PHA**

**Markos Petousis [a], Nikolaos Michailidis [b, c], Nikolaos Mountakis [a], Apostolos Argyros [b, c], Maria Spyridaki [a], Emmanuel Maravelakis [d], Nektarios Nasikas [e], Nectarios Vidakis [a*]**

[a] Department of Mechanical Engineering, Hellenic Mediterranean University, Heraklion 71410, Greece

[b] Physical Metallurgy Laboratory, Mechanical Engineering Department, School of Engineering, Aristotle University of Thessaloniki, 54124 Thessaloniki, Greece

[c] Centre for Research & Development of Advanced Materials (CERDAM), Center for Interdisciplinary Research and Innovation, Balkan Centre, Building B', 10th km Thessaloniki-Thermi road, 57001, Thessaloniki, Greece

[d] Department of Electronic Engineering, Hellenic Mediterranean University, Chania 73133, Greece

[e] Division of Mathematics and Engineering Sciences, Department of Military Sciences, Hellenic Army Academy, 16673 Vari, Attica, Greece

* Corresponding author, E-mail: vidakis@hmu.gr (Nectarios Vidakis), Tel.: +302810379227

**Abstract**

The wide range of materials featuring unique properties has contributed to the constant growth of 3D-printed items nowadays. Polyhydroxyalkanoate (PHA) is a bio-sourced material that is gradually growing in additive manufacturing. 3D printed PHA was examined herein under dynamic mechanical analysis. The aim was to reveal the critical 3D printing settings affecting the response of this eco-friendly polymer on its rheology and under combined thermal and force loadings, producing valuable information for the enrichment of the available experimental data. Optimization was attempted with Taguchi L9 experimental design, with four control parameters: deposition speed, layer height, extrusion temperature, and extrusion width. The response metrics were the Flexural Storage Modulus, Dynamic Glass Transition Temperature, and Damping Factor at Dynamic Glass Transition Temperature. Two regression models were applied and compared to form reliable prediction equations, and a confirmation run verified the outcome. Optical microscopy evaluated the samples' microstructure and quality. Two controls were distinguished for their remarkable impact, namely, deposition speed and layer height. Flexural Storage Modulus increased ~15% with optimized settings selection. The optimization significance is unequivocal, promoting the utilization of PHA in Additive Manufacturing, with the valuable information provided on the mechanical response of this nature-sourced polymer.

## S1. Design of experiments and modeling procedure

Initially, the Taguchi method was employed, with the aim of choosing cases for modeling from the orthogonal array. The purpose of its use is to avoid the complexity of classical experimental designs,

and the great number of simulations and experiments which would be necessary to be conducted as the printing parameters increase.

The utilization of the Taguchi design of experiment provided the ability to examine the entire parameter space, while fewer experiments are necessary to reach problem solution [1]. Normally, the orthogonal array is selected by utilizing the total degree of freedom (DOF), which is the number of factor levels minus 1, for each factor [2], and is supposed to be lower than the DOF of the chosen orthogonal array.

Continuing, Taguchi is used for the analysis of the fact that different modeled parameters can have an impact on the solution of the issue and receive the most suitable parameter combination [3]. Its proven favor [4–8] has led to it being employed in several research fields [9]. The method provides with putting in order independent parameters judging by their importance for dependent objective functions [10], while also a parameters set is created in such a way as to result in the best-case and worst-case scenarios.

For the calculation process signal-to-noise (S/N) ratios for all the factors under investigation, delta values from S/N ratios, and determination of the factor's order are calculated. The S/N ratio is defined in three forms: the larger is the better, the smaller is the better, and the nominal is the best [11]. These equations are listed below:
The larger the better [12]:

$$S/_{N} = -10 \log\left(\frac{1}{n}\sum_{i=1}^{n}\frac{1}{Y_i^2}\right) \tag{S1}$$

Smaller the better [4]:

$$S/_{N} = -10 \log\left(\frac{1}{n}\sum_{i=1}^{n}Y_i^2\right) \tag{S2}$$

Nominal the best [13,14]:

$$S/_{N} = 10 \log\left(\frac{\mu^2}{\sigma^2}\right) \tag{S3}$$

Where μ is for means and σ is for standard deviation, and Yi is for the resulting value for the $i_{th}$ objective function.

The maximum and minimum S/N values of all parameters' differences are used for the delta values calculation and then they are ranked. The most effective parameter is registered as the largest delta value and the rank results from this order.

ANOVA statistical technique is spread widely and often utilized, for interpretation of the experimental results derived by obtaining the contribution ratio of all parameters. It examines each parameter's

significance for the current problem in need of a solution for the problem related to DMA results. The calculation process of ANOVA is described below:

The total sum of squares ($SS_T$) was found according to Ref. [15]:

$$SS_T = \sum_{i=1}^{N} \left(Y_i - \overline{Y}\right)^2 \tag{S4}$$

Where N is the number of cases in the orthogonal array, $Y_i$ is the experimental/numerical result for the $i_{th}$ experiment,

$$\overline{Y} = \frac{1}{N}\sum_{i=1}^{N} Y_i \tag{S5}$$

The $SS_T$ consists of the sum of the squared error $SS_e$ and the sum of the squared deviations $SS_P$ due to each process parameter, therefore $SS_P$ was defined as [15]:

$$SS_P = \sum_{j=1}^{t} \frac{\left(SY_j\right)^2}{t} - \frac{1}{N}\left[\sum_{i=1}^{N} Y_i\right]^2 \tag{S6}$$

Where P is one of the parameters, j is the level number of P, t is the repetition of every level of P, and $SY_j$ is the sum of the experimental results involving P and level j. The sum of squares from the error parameters $SS_e$ is [15]:

$$SS_e = SS_T - SS_A - SS_B - SS_C - SS_D \tag{S7}$$

The total degree of freedom is $D_T = N - 1$, and the degree of freedom of each tested parameter is $D_P = N - 1$. The variance of the parameter tested is $V_P = SS_P/D_P$. Then, the F-value for every design parameter is simply the ratio of the mean of squares deviations to the mean of the squared error $F_P = V_P/V_e$. The percentage contribution ρ was calculated as [15]:

$$\rho_P = \frac{SS_P}{SS_T} \tag{S8}$$

The control parameters of the Taguchi L9 array optimization analysis herein were namely $D_S(\mathrm{mm/s})$, $L_H(\mathrm{mm})$, $E_T(°C)$ and $E_W(\%)$, each having three levels. On the other hand, the response metrics were $E^{F'}$, $DT_g$ and $DFT_g$.

There are two methods, the Linear Regression Model (LRM) and the Reduced Quadratic Regression Model (RQRM), which were employed in order to compare their efficiency for the present analysis.

The LRM for each response is calculated by:

$$Y_k = a_k + \sum_{i=1}^{n} b_{i,k} x_i + e_k \tag{S9}$$

The RQRM for each response is calculated by:

$$Y_k = a_k + \sum_{i=1}^{n} b_{i,k} x_i + \sum_{i=1}^{n} c_{i,k} x_i^2 + e_k \quad \text{(S10)}$$

Where k represents the response output (i.e., $E^{F'}$, $DT_g$, $DFDT_g$), a the constant value, b the coefficients of the linear terms, c the coefficients of the square terms, e the error and $x_i$ the four (n=4) control parameters (i.e. $D_S$, $L_H$, $E_T$, and $E_W$).

## S2. Experimental data

**Table S1**. Measured $E^{F'}$, $DT_g$, $DFT_g$ for each experimental run and five replicas per run

| **A/A** | **Run** | $\boldsymbol{E^{F'}(MPa)}$ | $\boldsymbol{DT_g(°C)}$ | $\boldsymbol{DFT_g}$ |
|---|---|---|---|---|
| 1 | | 2210.69 | 187.57 | 0.19 |
| 2 | 1 | 2336.93 | 192.77 | 0.18 |
| 3 | | 2244.36 | 190.22 | 0.19 |
| 6 | | 2213.52 | 188.35 | 0.18 |
| 7 | 2 | 2280.14 | 191.74 | 0.17 |
| 8 | | 2177.34 | 189.00 | 0.17 |
| 11 | | 2458.63 | 189.69 | 0.18 |
| 12 | 3 | 2435.33 | 192.76 | 0.18 |
| 13 | | 2444.21 | 191.98 | 0.18 |
| 16 | | 2186.23 | 179.72 | 0.19 |
| 17 | 4 | 2285.87 | 182.85 | 0.17 |
| 18 | | 2330.99 | 184.52 | 0.20 |
| 21 | | 2288.84 | 189.60 | 0.18 |
| 22 | 5 | 2337.33 | 189.86 | 0.19 |
| 23 | | 2222.04 | 195.07 | 0.19 |
| 26 | | 2031.10 | 186.42 | 0.18 |
| 27 | 6 | 2025.70 | 193.53 | 0.18 |
| 28 | | 2214.58 | 197.58 | 0.18 |
| 31 | | 2174.76 | 177.87 | 0.19 |
| 32 | 7 | 2153.47 | 192.17 | 0.18 |
| 33 | | 2368.43 | 188.55 | 0.18 |
| 36 | | 2165.41 | 186.30 | 0.19 |
| 37 | 8 | 2064.24 | 191.14 | 0.18 |
| 38 | | 2016.66 | 188.39 | 0.17 |
| 41 | | 2050.87 | 187.78 | 0.17 |
| 42 | 9 | 1941.95 | 191.62 | 0.17 |
| 43 | | 2253.55 | 188.51 | 0.17 |

| | | | | |
|---|---|---|---|---|
| | Min: | 1941.95 | 177.87 | 0.17 |
| | Max: | 2458.63 | 197.58 | 0.20 |
| | Average: | 2219.01 | 189.09 | 0.18 |

**Table S2**. Measured $E^{F'}$, $DT_g$, $DFT_g$ for the five replicas of the Confirmation experimental run

| A/A | Run | $E^{F'}(MPa)$ | $DT_g(°C)$ | $DFT_g$ |
|---|---|---|---|---|
| 1 | | 2329.97 | 187.99 | 0.17 |
| 2 | 10 | 2432.52 | 194.83 | 0.18 |
| 3 | | 2274.55 | 190.88 | 0.17 |
| | Min: | 2274.55 | 187.99 | 0.17 |
| | Max: | 2432.52 | 194.83 | 0.18 |
| | Average: | 2345.68 | 191.23 | 0.17 |

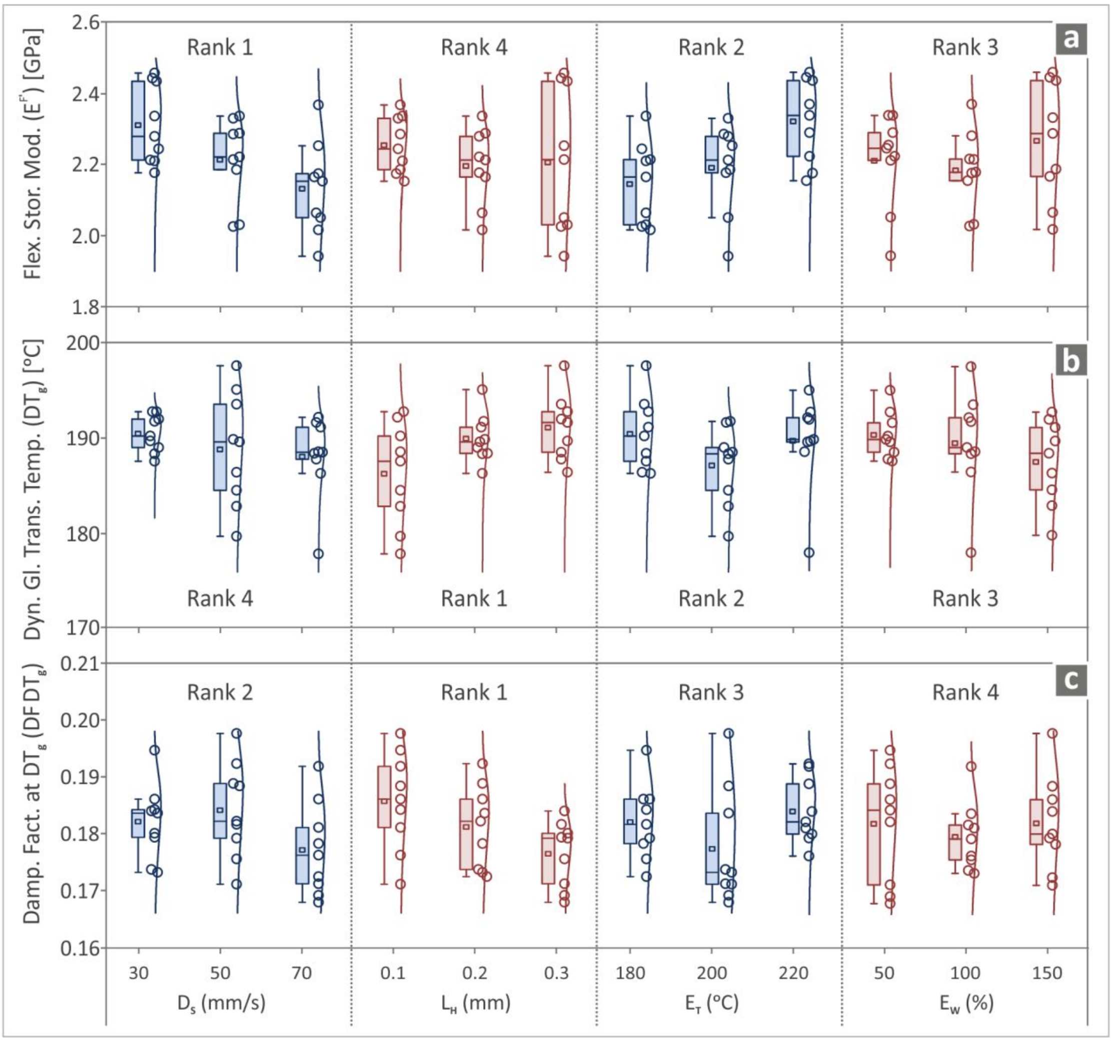


**Figure S1.** Box plots and ranks, considering (a) $E^{F'}$, (b) $DT_g$, and (c) $DFT_g$

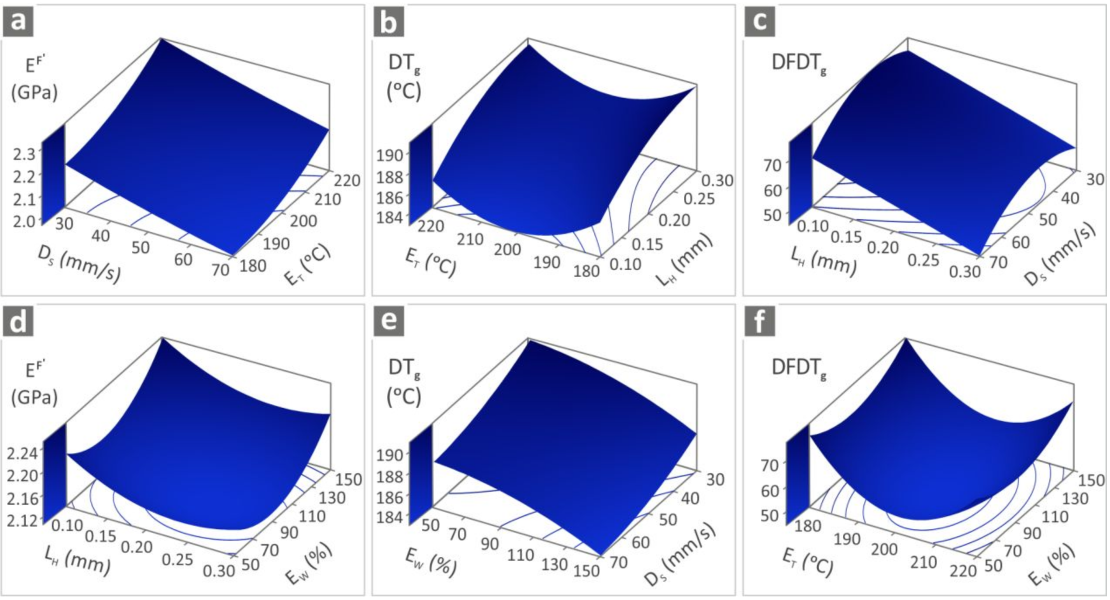


**Figure S2.** Surface 3D plots showing (a-c) Rank 1 and 2 and (d-f) Rank 3 and 4 for each response

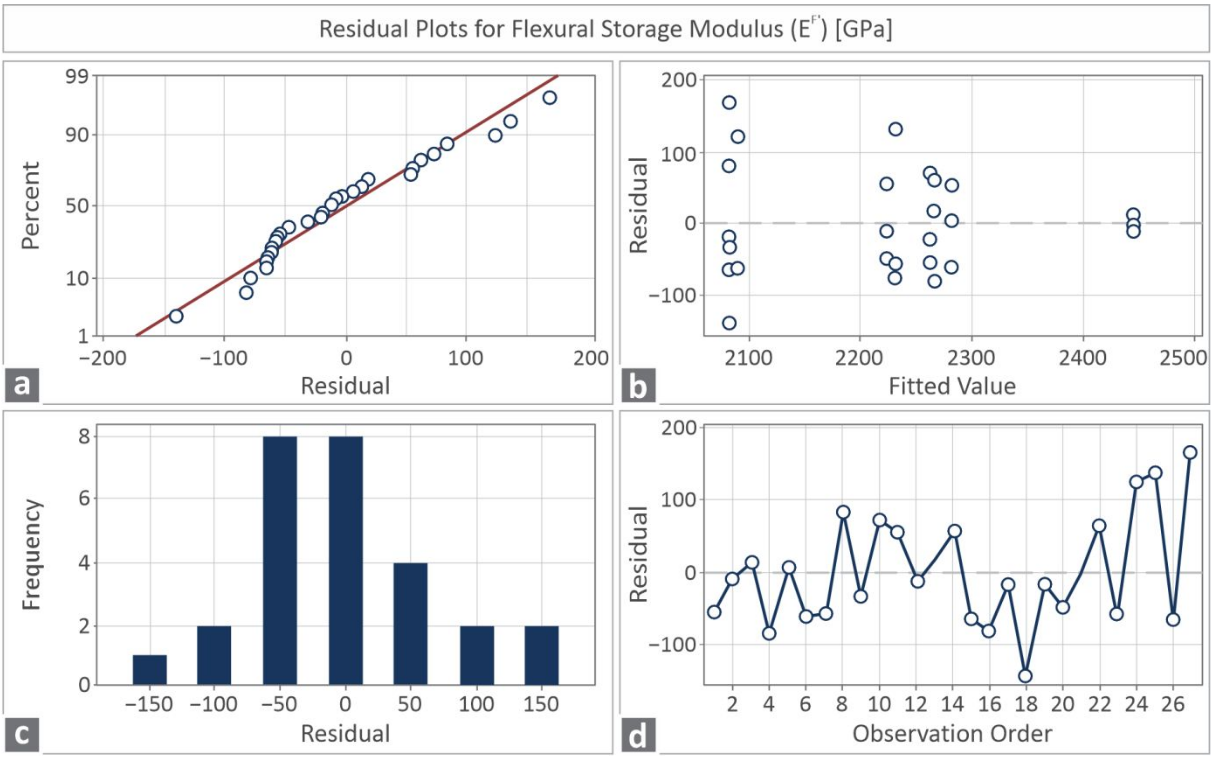


**Figure S3.** Residual plots for the Flexural Storage Modulus (a) Percent vs. Residual, (b) Residual vs. Fitted Value, (c) Frequency vs. Residual, (d) Residual vs. Observation Order

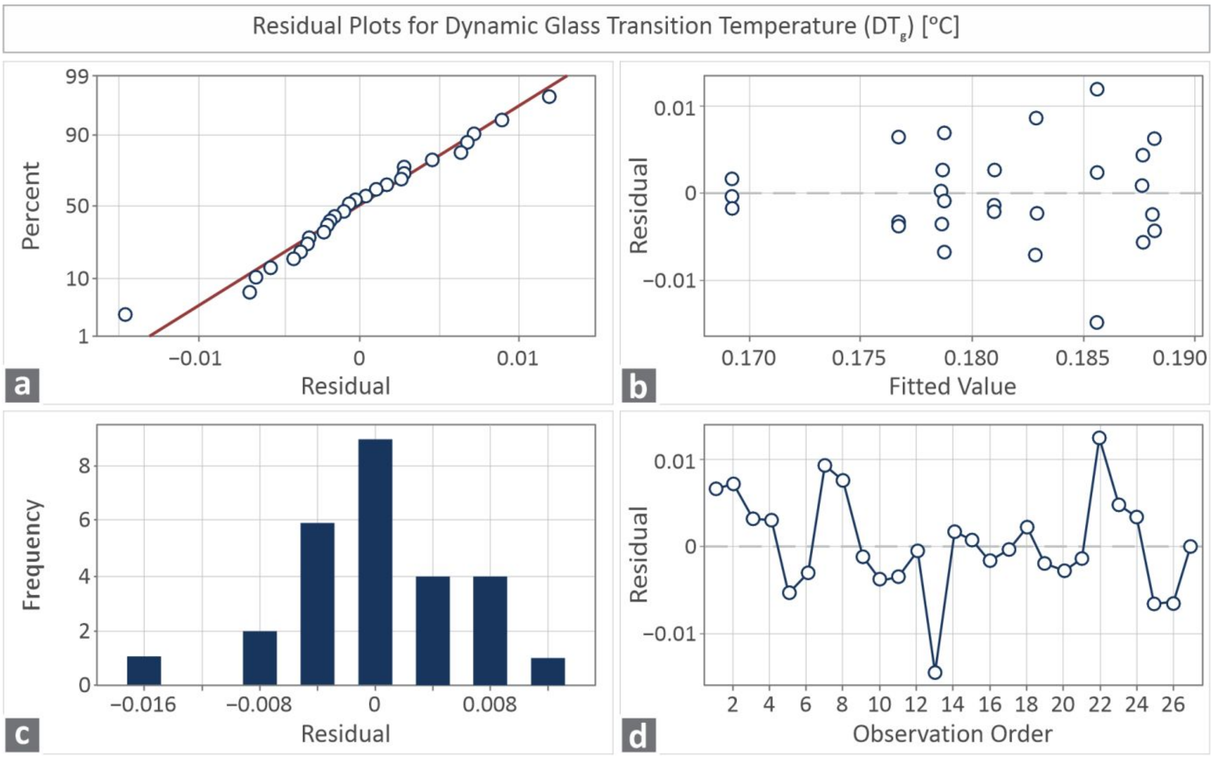


**Figure S4.** Residual plots for the Glass Transition Temperature (a) Percent vs. Residual, (b) Residual vs. Fitted Value, (c) Frequency vs. Residual, (d) Residual vs. Observation Order

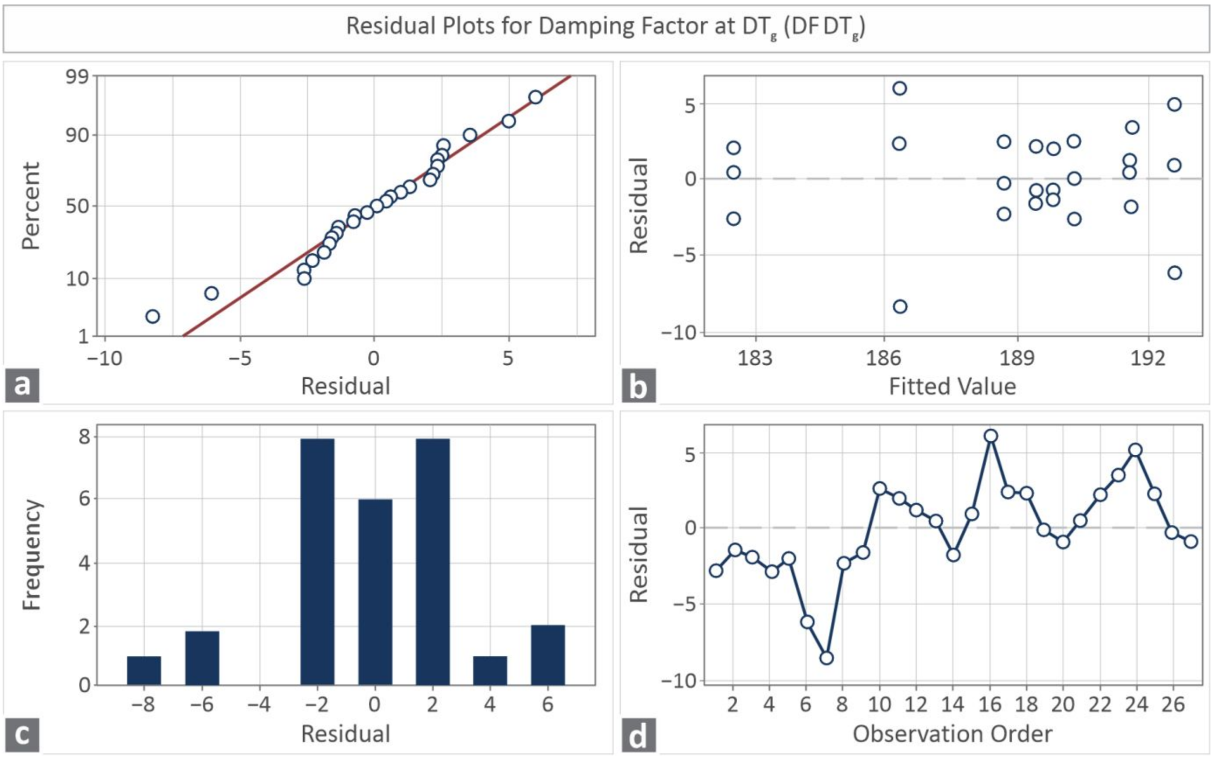


**Figure S5.** Residual plots for the Damping Factor (a) Percent vs. Residual, (b) Residual vs. Fitted Value, (c) Frequency vs. Residual, (d) Residual vs. Observation Order

**References**

(1) Yang, W. H.; Tarng, Y. S. Design Optimization of Cutting Parameters for Turning Operations Based on the Taguchi Method. *J Mater Process Technol* **1998**, *84* (1–3), 122–129. https://doi.org/10.1016/S0924-0136(98)00079-X.

(2) Ning, M.; Mengjie, S.; Mingyin, C.; Dongmei, P.; Shiming, D. Computational Fluid Dynamics (CFD) Modelling of Air Flow Field, Mean Age of Air and CO2 Distributions inside a Bedroom with Different Heights of Conditioned Air Supply Outlet. *Appl Energy* **2016**, *164*, 906–915.

(3) Niu, B.; Shi, M.; Zhang, Z.; Li, Y.; Cao, Y.; Pan, S. Multi-Objective Optimization of Supply Air Jet Enhancing Airflow Uniformity in Data Center with Taguchi-Based Grey Relational Analysis. *Build Environ* **2022**, *208*, 108606. https://doi.org/10.1016/j.buildenv.2021.108606.

(4) Arslanoglu, N.; Yigit, A. Experimental Investigation of Radiation Effect on Human Thermal Comfort by Taguchi Method. *Appl Therm Eng* **2016**, *92*, 18–23. https://doi.org/10.1016/j.applthermaleng.2015.09.070.

(5) Chang, C.-W.; Kuo, C.-P. Evaluation of Surface Roughness in Laser-Assisted Machining of Aluminum Oxide Ceramics with Taguchi Method. *Int J Mach Tools Manuf* **2007**, *47* (1), 141–147. https://doi.org/10.1016/j.ijmachtools.2006.02.009.

(6) Pinar, A. M.; Uluer, O.; Kırmaci, V. Optimization of Counter Flow Ranque–Hilsch Vortex Tube Performance Using Taguchi Method. *International Journal of Refrigeration* **2009**, *32* (6), 1487–1494. https://doi.org/10.1016/j.ijrefrig.2009.02.018.

(7) Özel, S.; Vural, E.; Binici, M. Optimization of the Effect of Thermal Barrier Coating (TBC) on Diesel Engine Performance by Taguchi Method. *Fuel* **2020**, *263*.

(8) Tutar, M.; Aydin, H.; Yuce, C.; Yavuz, N.; Bayram, A. The Optimisation of Process Parameters for Friction Stir Spot-Welded AA3003-H12 Aluminium Alloy Using a Taguchi Orthogonal Array. *Mater Des* **2014**, *63*, 789–797. https://doi.org/10.1016/j.matdes.2014.07.003.

(9) G. Taguchi. Introduction to Quality Engineering, Asian Productivity Organization, Tokyo. **1990**.

(10) Arslanoglu, N.; Yigit, A. Investigation of Efficient Parameters on Optimum Insulation Thickness Based on Theoretical-Taguchi Combined Method. *Environ Prog Sustain Energy* **2017**, *36* (6), 1824–1831. https://doi.org/10.1002/ep.12628.

(11) P.J. Ross. Taguchi Techniques for Quality Engineering, Second, McGraw Hill, New York. **1996**.

(12) Bademlioglu, A. H.; Canbolat, A. S.; Yamankaradeniz, N.; Kaynakli, O. Investigation of Parameters Affecting Organic Rankine Cycle Efficiency by Using Taguchi and ANOVA Methods. *Appl Therm Eng* **2018**, *145*, 221–228. https://doi.org/10.1016/j.applthermaleng.2018.09.032.

(13) Soni, A.; Patel, R. M.; Kumar, K.; Pareek, K. Optimization for Maximum Extraction of Solder from Waste PCBs through Grey Relational Analysis and Taguchi Technique. *Miner Eng* **2022**, *175*, 107294. https://doi.org/10.1016/j.mineng.2021.107294.

(14) Palanikumar, K. Experimental Investigation and Optimisation in Drilling of GFRP Composites. *Measurement* **2011**, *44* (10), 2138–2148. https://doi.org/10.1016/j.measurement.2011.07.023.

(15) Vidal, C.; Infante, V.; Peças, P.; Vilaça, P. Application of Taguchi Method in the Optimization of Friction Stir Welding Parameters of an Aeronautic Aluminium Alloy. *International Journal of Advanced Materials Manufacturing and Characterization* **2013**, *3* (1), 21–26. https://doi.org/10.11127/ijammc.2013.02.005.
